# Predicting future thermal habitat suitability of competing native and invasive fish species: from metabolic scope to oceanographic modelling

**DOI:** 10.1093/conphys/cou059

**Published:** 2015-01-20

**Authors:** Stefano Marras, Andrea Cucco, Fabio Antognarelli, Ernesto Azzurro, Marco Milazzo, Michel Bariche, Momme Butenschön, Susan Kay, Massimiliano Di Bitetto, Giovanni Quattrocchi, Matteo Sinerchia, Paolo Domenici

**Affiliations:** 1 IAMC-CNR, Institute for the Coastal Marine Environment, National Research Council, Localitá Sa Mardini, Torregrande, OR 09170, Italy; 2 Institute for Environmental Protection and Research (ISPRA), Piazzale dei Marmi 2, Livorno 57123, Italy; 3 Dipartimento di Scienze Della Terra e del Mare, University of Palermo, via Archirafi 28, Palermo 90123, Italy; 4 Department of Biology, FAS, American University of Beirut, Riad El-Solh, Beirut 1107 2020, Lebanon; 5 Plymouth Marine Laboratory, Prospect Place, The Hoe, Plymouth PL1 3DH, UK; 6 Central Management for Programming and Infrastructures, National Research Council, Piazzale Aldo Moro 7, Roma 00185, Italy

**Keywords:** Conservation physiology, distribution modelling, fish physiology, global warming, invasive species, thermal habitat suitability

## Abstract

Global increase in sea temperatures has been suggested to facilitate the incoming and spread of tropical invaders. The increasing success of these species may be related to their higher physiological performance compared with indigenous ones. Here, we determined the effect of temperature on the aerobic metabolic scope (MS) of two herbivorous fish species that occupy a similar ecological niche in the Mediterranean Sea: the native salema (*Sarpa salpa*) and the invasive marbled spinefoot (*Siganus rivulatus*). Our results demonstrate a large difference in the optimal temperature for aerobic scope between the salema (21.8°C) and the marbled spinefoot (29.1°C), highlighting the importance of temperature in determining the energy availability and, potentially, the distribution patterns of the two species. A modelling approach based on a present-day projection and a future scenario for oceanographic conditions was used to make predictions about the thermal habitat suitability (THS, an index based on the relationship between MS and temperature) of the two species, both at the basin level (the whole Mediterranean Sea) and at the regional level (the Sicilian Channel, a key area for the inflow of invasive species from the Eastern to the Western Mediterranean Sea). For the present-day projection, our basin-scale model shows higher THS of the marbled spinefoot than the salema in the Eastern compared with the Western Mediterranean Sea. However, by 2050, the THS of the marbled spinefoot is predicted to increase throughout the whole Mediterranean Sea, causing its westward expansion. Nevertheless, the regional-scale model suggests that the future thermal conditions of Western Sicily will remain relatively unsuitable for the invasive species and could act as a barrier for its spread westward. We suggest that metabolic scope can be used as a tool to evaluate the potential invasiveness of alien species and the resilience to global warming of native species.

## Abstract (precis)

Global increase in sea temperatures has been suggested to facilitate the incoming and spread of tropical invaders. Here, we determined the effect of temperature on the aerobic metabolic scope of two competing fish species, one native and one invasive, and we predicted their future thermal habitat suitability.

## Introduction

Sea surface temperatures are predicted to continue rising globally throughout the 21st century, with dramatic consequences for marine ecosystems in the near future (IPCC, 2013). Rapid poleward shifts of isotherms were recorded in recent decades, specifically in the northern hemisphere (Burrows *et al.*, 2011), ultimately contributing to changes in the composition of marine communities (Harley *et al.*, 2006). Direct effects of temperature changes include a shift in species distribution (i.e. range contractions or extensions) towards higher latitudes and depths (Perry *et al.*, 2005) and an increase in extinction rates of many marine organisms (Ben Rais Lasram *et al.*, 2010). It was argued that one of the first processes to cause extinction or relocation to cooler waters is the reduction in aerobic performance in warming conditions (Pörtner and Knust, 2007). However, species do not exist in isolation but as part of large communities of interacting species; recent studies revealed the importance of taking into account species interactions as biotic multipliers of climate change effects (Zarnetske *et al.*, 2012; Blois *et al.*, 2013; Milazzo *et al.*, 2013). Therefore, the relative changes in the performance of interacting species are expected to provide useful insights to explain invasion success (Azzurro *et al.*, 2014) and to predict future changes in species distribution (Pörtner and Knust, 2007; Pörtner and Farrell, 2008).

In the Mediterranean Sea, warming conditions are believed to be facilitating the arrival and spread of tropical invaders at an unprecedented rate (Hiddink *et al.*, 2012). This basin can be considered as a model area for investigation of the different thermal windows of interacting species and the potential ecological consequences of climate change. At present, almost 90 fish species (Belmaker *et al.*, 2013; Golani *et al.*, 2002) coming from the Red Sea (hereafter referred as Lessepsian migrants) have entered the Mediterranean Sea through the Suez Canal. This represents the main invasion route for alien species (Galil, 2009; Zenetos *et al.*, 2010, 2012). This phenomenon, once confined to Levantine waters (Por, 1990), is proceeding with an apparent accelerating rate of introductions (Galil, 2009). Moreover, it has recently started to extend westwards through the Sicilian Channel (Quignard and Tomasini, 2000), which separates the western and the eastern sectors of the basin. This channel has long been considered the ultimate limit to the spread of Lessepsian fish species to the Western Mediterranean (Quignard and Tomasini, 2000). However, in the last two decades, the number of tropical and subtropical fishes recorded in this area has dramatically increased (Azzurro *et al.*, 2013a), and a few of them recently reached the western sectors of the basin (e.g. *Fistularia commersonii*, Azzurro *et al.*, 2012). An increasing number of studies have used ecological modelling for predicting the future distribution of native and invasive fishes in the Mediterranean Sea (Ben Rais Lasram *et al*., 2010; Albouy *et al*., 2012, 2013) and have suggested that the projected climate change might cause a significant loss of climatically suitable habitat for endemic fish species (Albouy *et al.*, 2013).

The increasing success of the tropical invasive species may be related in part to the fact that their thermal window of aerobic performance is likely to be shifted towards warmer temperatures than that of the temperate indigenous species. Here, we investigated the relationship between temperature and aerobic performance in a pair of herbivorous fish species that occupy a similar ecological niche: the invasive marbled spinefoot (*Siganus rivulatus*) and its native counterpart, the salema (*Sarpa salpa*). These two species are among the few herbivorous fishes occurring in the Mediterranean (Azzurro *et al.*, 2007) and they may compete with each other (Golani, 1993), especially in conditions of limiting trophic resources, such as reduced algal biomass (Sala *et al.*, 2011). The marbled spinefoot entered the Mediterranean through the Suez Canal in the 1920s and established a large population along the African coast around the 1940s (Golani *et al.*, 2002). It has also spread through the Sicilian Channel, via the African coasts (Golani *et al.*, 2002), but has not yet entered the Western Mediterranean (Zenetos *et al.*, 2010; Golani*, et al.*, 2002). The native salema is very common and widespread along the Mediterranean coasts (Verlaque, 1990), but in the easternmost sectors of the Mediterranean, this species has probably been outcompeted by the marbled spinefoot (Bariche *et al.*, 2004; Galil, 2009), and a competitive superiority of marbled spinefoot vs. salema has been hypothesized (Bariche *et al.*, 2004). Furthermore, other Siganid species (e.g. *Siganus luridus*) have recently extended their distribution, colonizing the islands of the Sicilian Channel (Schembri *et al.*, 2012; Azzurro and Andaloro, 2014). Expected changes in the relative dominance of these two herbivorous species might have important ecological consequences in the near future, because Siganids have the potential to graze algal resources intensively and cause large-scale impact on natural habitats and local food chains (Sala *et al.*, 2011; Vergés *et al.*, 2014a).

**Figure 1:**
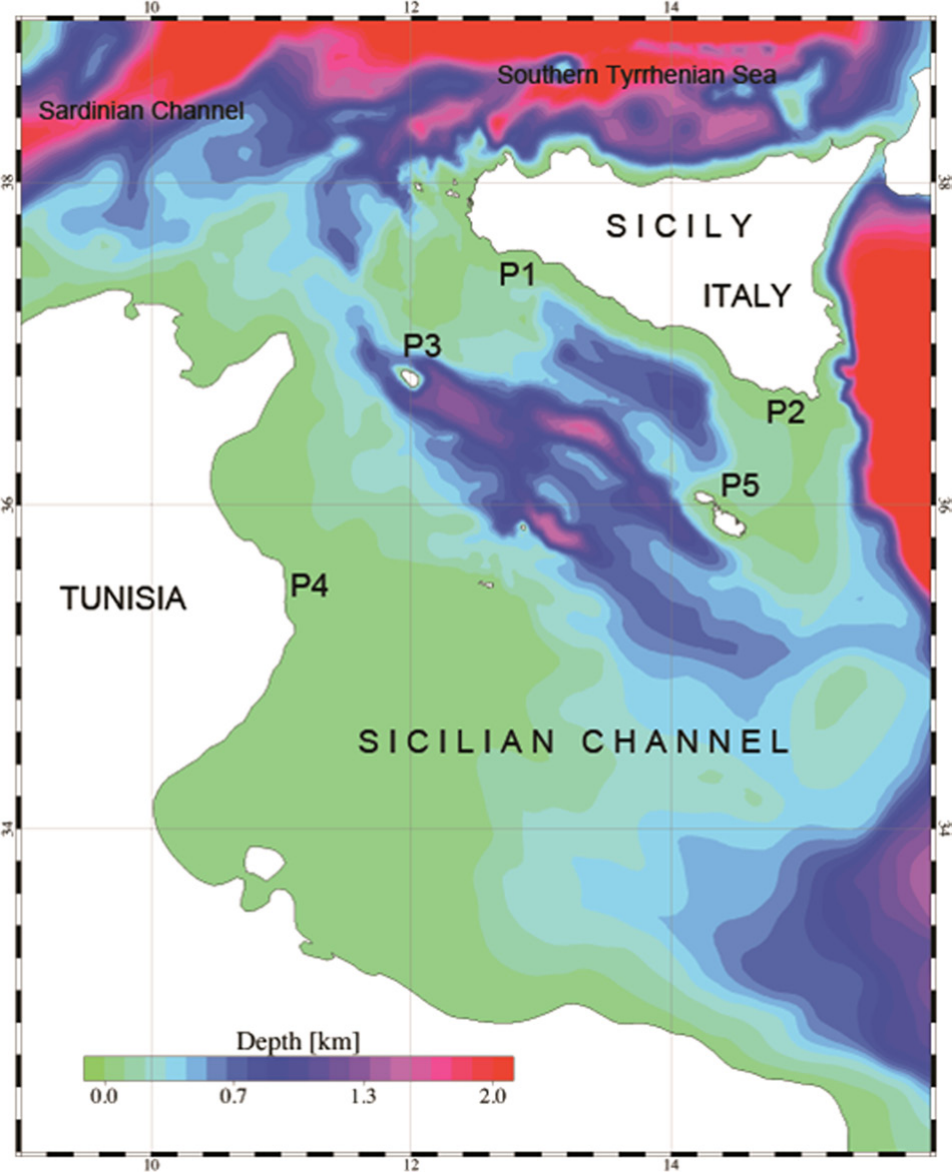
Sicilian Channel, study area. P1 to P5 show the locations of the stations adopted for a time series analysis of the NMS in the regional-scale model.

Here, we aim to estimate how changes in water temperature influence the physiological performances (i.e. metabolic scope, MS) of both marbled spinefoot and salema*.* Metabolic scope represents the scope within which aerobic activities, such as swimming, feeding or growth, must be accommodated (Fry, 1971). As such, MS is a suitable metric for analysing the energetics of habitat selection (Fry, 1971) and is related to the ecological success of a given species (Pörtner and Knust, 2007). Thus, MS can provide a bioenergetic basis for studying how environmental constraints govern the spatial and temporal distribution pattern of fishes (Cucco *et al.*, 2012a). We used this information to model the thermal habitat suitability (THS, a dimensionless index based on the relationship between MS and temperature) in relationship to current and future environmental conditions, taking the whole Mediterranean Sea into account, and focusing on the Sicilian Channel (Fig. 1) because of its biogeographical relevance for the westward progress of this invasion. A multidisciplinary approach combining a physiological empirical model and oceanographic numerical models was employed to evaluate THS of marbled spinefoot vs. salema, and to allow predictions of future fish distributions.

## Materials and methods

### Area of investigation

The Mediterranean Sea is divided in two main sub-sectors, the western and the eastern basins, which are connected through the shallower Sicilian Channel (Manzella *et al.*, 1988; Astraldi *et al.*, 1999). The exchanges of water with the Atlantic Ocean, the Black Sea and the Red Sea are controlled through the shallow straits of Gibraltar, the Bosphorus and the Suez Channel, respectively. The average surface temperature ranges roughly between 16 and 20°C in the winter and between 24 and 28°C in the summer, with generally higher temperatures (by ∼2°C) in the eastern basin. Mediterranean isotherms are rapidly shifting west- and northwards, and average sea surface temperature is predicted to increase by a further 2.3–2.6°C or even 3.1°C by the end of this century (Somot *et al.*, 2008).

The water circulation of the Sicilian Channel is characterized by the presence of two main current patterns: a south-eastern surface flow, which transfers the Atlantic water (AW) from the western to the eastern sub-basin; and a north-westward sub-surface current in the opposite direction, which exchanges the intermediate Levantine water (LIW) between the eastern and western sub-basins (Malanotte-Rizzoli, 2001). Ten years (1983–1992) of monthly averaged sea surface temperature maps reveal that during the climatological winter, the isotherms run zonally from the Sicilian Channel to Cyprus. This generates a bimodal (north–south oriented) regimen of the surface temperature within the Sicilian Channel (Marullo *et al.*, 2007). The climatological summer yields southern thermal fronts, especially within the Sicilian Channel, where a strong coastal upwelling appears along the Sicilian coastlines. Two regions with different thermal regimens dominate the Sicilian Channel throughout the year: a warmer region along the African coasts and a colder region along the southern coasts of Sicily.

### Overview of methods

Physiological empirical models and oceanographic numerical modelling were used to make predictions of how changes in environmental conditions may affect the thermal habitat suitability of the native salema and the invasive marbled spinefoot in the Mediterranean Sea. Two different climate scenarios were considered: a present-day projection (RA, hereafter ‘present day’) corresponding to the past decade from 2001 to 2009; and a near future scenario, 2040–2049, based on a national enterprise scenario driven by atmospheric conditions of an A2 AR4 SRES projection (A2, hereafter ‘future scenario’). The overall approach consists of the following steps: (i) measurement of metabolic scope in the two fish species and defining the equation relating MS and temperature; (ii) modelling the oceanographic conditions to predict the changes in temperature and other hydrodynamic variables in the selected coastal waters in relationship to the present-day projection and the future scenarios; and (iii) coupling the physiological relationships with the oceanographic model to run the simulations of the present-day projection and the future scenarios.

### Measuring the metabolic scope of salema and marbled spinefoot

A total of 72 wild juvenile marbled spinefoot (total length 11.8 ± 1.3 cm; mass 19.1 ± 6.4 g at the time of testing) were captured along the Lebanese coast, offshore of Beirut. They were transported via air cargo to the IAMC-CNR laboratory in Oristano (Sardinia), where they were transferred to a circular tank (1.5 m diameter) supplied with recirculated natural sea water maintained at constant temperature (20 ± 0.3°C) and salinity (35.1 ± 0.2‰), under a natural photoperiod. A total of 72 wild juvenile salema (total length 11.1 ± 1.1 cm; mass 19.4 ± 5.8 g at the time of testing) were captured in the Oristano Gulf, Sardinia, Italy and transported to the IAMC-CNR laboratory, where they were maintained in the same way as the marbled spinefoot. One month later, groups of 12 individuals of each species were transferred to rectangular 100 l aquaria (a total of 12 aquaria, six per species, each one containing 12 individuals). The six different aquaria for each species were used to acclimate each group of 12 fish to six different water temperatures (i.e. 17, 20, 23, 26, 29 and 32°C). Final acclimation temperature was reached by increasing (or decreasing, depending of the final acclimation temperature) the water temperature by 1°C per day. Fish were left for 1 month at the final acclimation temperature before a single experiment. Note that none of *S. salpa* individuals survived at acclimation temperatures higher than 26°C. Fish were fed daily with commercial pellets, and feeding was suspended for at least 24 h before testing.

Metabolic rate was measured in individual animals. Fish were removed from the holding tank and introduced into a round tank (50 l), where they were chased to exhaustion. Fish were considered exhausted when they did not respond to stimulation (Roche *et al.*, 2013). Immediately after exhaustion, fish were placed in one of four static respirometers (0.5 l; Loligo Systems, Tjele, Denmark) immersed in an outer tank. Instantaneous oxygen uptake (*M*O_2_, in milligrams of O_2_ per kilogram per hour) was measured by intermittent flow respirometry (Steffensen, 1989) once every 30 min. Temperature was kept constant for the duration of the experiment. Metabolic scope was assessed in non-limiting oxygen conditions (i.e. in normoxia corresponding to an oxygen saturation >80%). Water flow from the external bath through the respirometers was driven by an external pump that was set to turn on and off for alternating 15 min periods. This allowed decreases in water oxygen content to be measured every 15 s for 15 min while the respirometer was in the closed state. The respirometer was then flushed with aerated water for 15 min. The oxygen consumption during each closed phase was calculated using linear least-squares regression (excluding the first and last 2 min of each closed phase). Fish could not see each other nor interact while in the separate respirometers during the measurement of oxygen consumption. Water oxygen levels were measured with optodes (Oxy-4 mini; PreSens Precision Sensing GmbH, Regensburg, Germany) and associated software (Pre-Sens Oxy 4v2), and *M*O_2_ was then calculated (Marras *et al.*, 2010, 2013; Killen *et al.*, 2011, 2012). Fish were left in the tank for 24 h, then removed, measured for length, width and weight and placed in a common holding tank different from the one used for acclimation. Measurements of background microbial respiration in the system were taken before and after the oxygen measurement in the respirometers.

The first 15 min (excluding the first and last 2 min as described above) of the oxygen consumption measurements obtained immediately after the introduction of individual fish into the respirometer were used to determine the maximal metabolic rate (MMR), which is the maximal aerobic metabolic rate measured in non-limiting conditions. Standard metabolic rate (SMR), which supports maintenance activities, such as ventilation or osmoregulation, and corresponds to the oxygen consumption of a resting, fasted and non-maturing fish, was measured using the 15th percentile method (Chabot and Claireaux, 2008). Metabolic scope (MS, i.e. the energy available to an individual to accomplish all of its tasks, including swimming, digestion etc.) was obtained as the difference between MMR and SMR as follows:

(1) MS=MMR−SMR,

where MMR (in milligrams of O_2_ per kilogram per hour) and SMR (in milligrams of O_2_ per kilogram per hour) are based on the following equations:

(2) $$\text{MMR}=d{\left(\frac{T_{m}-T}{T_{m}-T_{\mathrm{act}}}\right)}^{\omega }\times \exp\left[-\omega \left(\frac{T_{m}-T}{T_{m}-T_{\mathrm{act}}}\right)\right]$$

(3) SMR=a×exp⁡(b×T)

where *T*_act_ is the temperature at which MMR (*M*O_2max_ in the study by Lefrançois and Claireaux, 2003) is maximized and *T*_m_ is the temperature at which MMR is equal to zero. The values for *T*_act_, *T*_m_ and the remaining empirical parameters (*a*, *b*, *ω* and *δ*) were obtained as the result of the fitting procedure between the MMR, SMR experimental data and the parametric functions described by equations (2) and (3).

### The numerical models and the simulation set-up for present-day and future scenarios

Two different numerical approaches were followed to simulate the changes of the thermal habitat between the present-day projection and the future scenario at Mediterranean basin scale and at sub-regional scale for the Sicilian Channel area. At the basin scale, the Proudman Oceanographic Laboratory Coastal Ocean Modelling System [POLCOMS; Holt and James, 2001) was implemented. This model is a three-dimensional, baroclinic, primitive equations model using terrain following σ-coordinates in the vertical direction. The implementation of the Mediterranean Sea model extends over the whole basin, with an open boundary at the Gibraltar Strait and a closed boundary at the Bosphorus Strait. The model domain was discretized using a finite difference mesh with a spatial resolution of 0.1° for the horizontal directions and 40 levels with a variable intralevel distance for the vertical direction. At the regional scale, a high-resolution three-dimensional hydrodynamic numerical model (SHYFEM3D, Umgiesser and Bergamasco, 1995; Umgiesser *et al.*, 2004; Cucco *et al.*, 2009, 2012b) based on the finite element method was applied. SHYFEM3D solves the shallow water equations integrated over each layer in their formulations with water levels and transports. SHYFEM3D uses finite elements for horizontal spatial discretization, with *z*-layers for vertical discretization. The SHYFEM3D implementation covers the area comprised between 8.5° and 16.5° longitude and 31.2° and 39.5° latitude and includes the whole Sicilian Channel, part of the Sardinian Channel and Southern Tyrrhenian Sea. The model domain was discretized by an unstructured finite element mesh with a spatial resolution varying between 300 m along the coastlines and 10 km for the inner part of the channel and for the eastern and western domain boundaries. Forty levels were used for the vertical direction.

The data providing atmospheric and ocean boundary condition forcing of the POLCOMS model simulations were based on present-day reanalysis data, modified by reference to data from the ECHAM5 GCM for the future projections (Jungclaus *et al.*, 2006; Roeckner *et al.*, 2006). For the present-day model runs (2000–2009), atmospheric reanalysis data were taken from ERA-Interim data (http://www.ecmwf.int/en/research/climate-reanalysis/era-interim) for the meteorological forcing and from GLORYS2 reanalysis (www.mercator-ocean.fr) for the ocean boundary conditions. The spatial resolution is about 0.7° for ERA-Interim and 0.25° for GLORYS2, with a temporal resolution of at least 24 h for all variables. River flow data were taken from the Global *NEWS* database (Seitzinger *et al.*, 2005). A delta method was used to create forcing data for the future scenario, in order to retain the spatial and temporal resolution of the reanalysis data. The future scenario data set was generated from the present-day values by applying a modification of the same size as the difference between the 2000s and 2040s given by the GCM. Changes in river forcings were derived from the results of the ELME project (Langmead *et al.*, 2007).

A downscaling technique was adopted to simulate the hydrodynamics of the Sicilian Channel for the two decades of the present-day projection and future scenario. The approach consisted of using the three-dimensional oceanographic data [water temperature (*T*), water salinity (*S*), water currents (Vel) and water levels (η)] produced by the POLCOMS model basin-scale application, as well as the meteorological data sets previously described, as open boundaries and forcing conditions for the SHYFEM3D model runs. A relaxation procedure was adopted to constrain the SHYFEM3D model solution to the imposed *T*, *S* and Vel values along the whole lateral boundary and for an inner buffer zone extending 25 km inside the domain (Cucco *et al.*, 2012b). No river loads were included as lateral boundary conditions. This is because the investigated area is characterized by an arid and semi-arid climate, with no major river inflows.

### Coupling physiological relationships with modelling to assess thermal habitat suitability

The results of the present-day and future model runs were processed to obtain the temporal and spatial variability of the daily water temperature obtained by averaging the values computed between the sea surface and a depth of 30 m for the sub-regional scale analysis, and only at the surface for the basin-scale analysis. The data sets obtained consisted of horizontal distributions of the daily temperature values during the two climatological conditions computed both from the basin-scale model results and from the sub-regional model results. The temperature values were used as input data for computing the spatial and temporal variability of the MS of the two fish species for the present-day projection and the future scenario at both basin and regional scales. The mean monthly temperatures for the present-day projection and the future scenario in the Sicilian Channel ranged between 13.1 and 31.2°C.

The differences in the aerobic performance for a defined thermal environment between the two fish species were computed by defining the thermal habitat suitability (THS) and the coexistence factor (CF). The THS is a dimensionless index that was computed, for each element of the model domain, as the time average of the MS values normalized with respect to the maximal computed value *MS*_max_ (NMS):

(4) $${\text{THS}}_{i}=\frac{\sum_{t=0}^{N}({\text{MS}}_{i,t})}{N\times {\text{MS}}_{\max}}.$$

The THS computed for the whole simulated period (*N*) and for both fish species ranged between zero and one, providing a degree of suitability within each element of the model domain (*i*) in relationship to the two fish species.

Considering NMS:

(5) $${\text{NMS}}_{i}=\frac{{\text{MS}}_{i}}{{\text{MS}}_{\max}},$$

computed for each element of the domain and for both fish species, a theoretical threshold (*T*_MS_) of NMS for high aerobic performance was identified based on the current distribution of the two fish species and the corresponding thermal habitat suitability values. The coexistence factor was calculated from the QMS, which is a binary quantity calculated by associating to the elements *i* a value of one when THS > *T*_MS_ and a value of zero when NMS < *T*_MS_. The QMS provided a semi-quantitative evaluation of the environmental fitness for both fishes. The coexistence factor was defined as the difference between the QMS computed for the marbled spinefoot and those computed for the salema. Coexistence factor ranges between minus one and one and reads as:

(6) $${\text{CF}}_{i}={\text{QMS}}_{i}^{S}-{\text{QMS}}_{i}^{R},$$

$$\begin{array}{ll} & {\text{QMS}}_{i}^{S}=0\;{\text{THS}}_{i}\leq T_{\mathrm{MS}},\\ & {\text{QMS}}_{i}^{S}=1\;{\text{THS}}_{i}>T_{\mathrm{MS}},\\ & {\text{QMS}}_{i}^{R}=0\;{\text{THS}}_{i}\leq T_{\mathrm{MS}},\\ & {\text{QMS}}_{i}^{R}=1\;{\text{THS}}_{i}>T_{\mathrm{MS}}, \end{array}$$

where the superscripts *R* and *S* stand for marbled spinefoot and salema, respectively. A negative and a positive value of CF indicate environmental conditions favouring marbled spinefoot and salema, respectively, whereas CF = 0 indicates areas where fish species are favoured equally by the thermal conditions.

## Results

### Metabolic response of salema and marbled spinefoot

**Figure 2:**
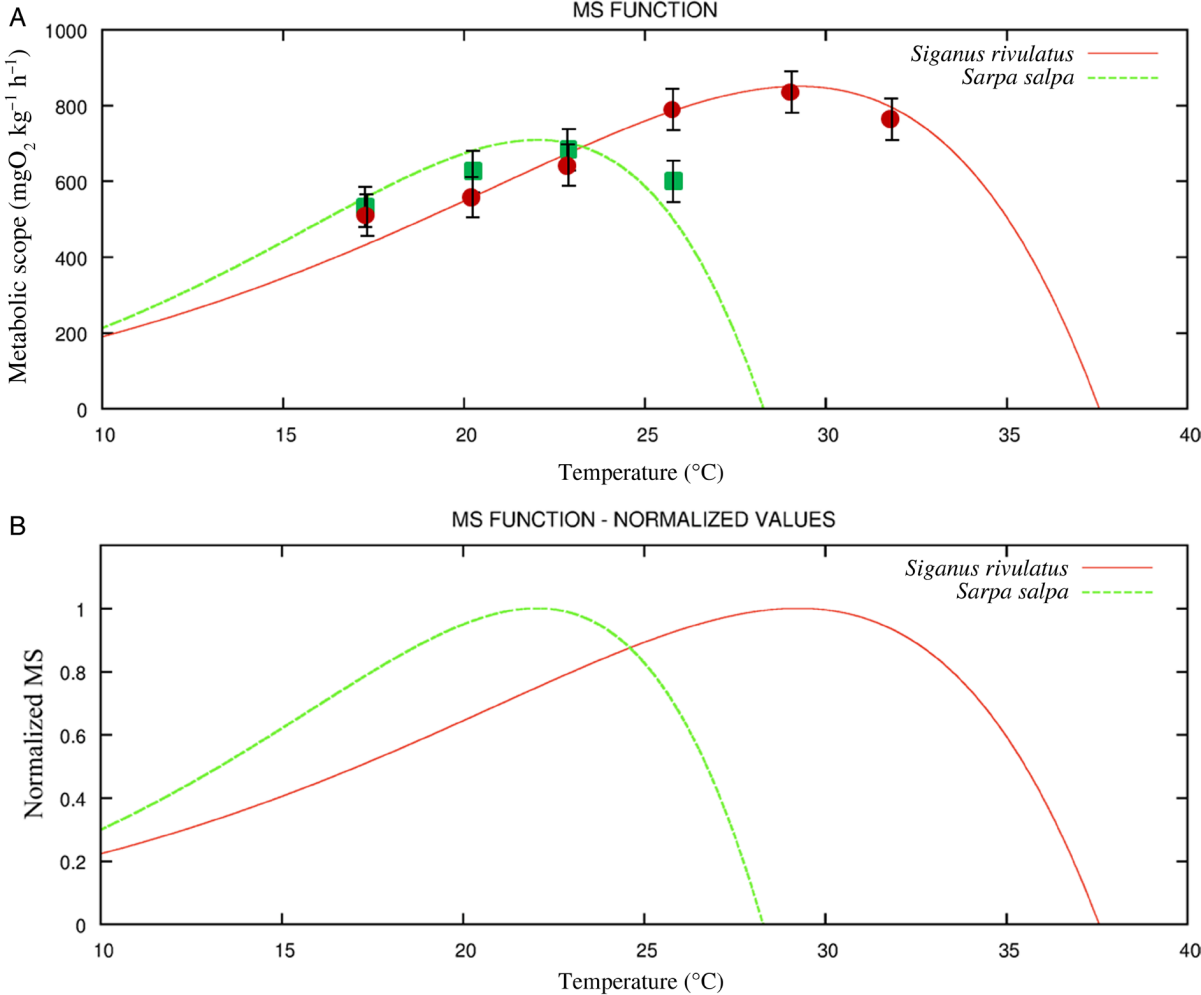
(A) Metabolic scope (MS) curve as a function of temperature in marbled spinefoot (*Siganus rivulatus*) and salema (*Sarpa salpa*). Green squares represent the MS values measured in salema and red circles represent the MS values measured in marbled spinefoot (means ± SD). Note that none of the *S. salpa* individuals survived at acclimation temperatures higher than 26°C. (B) Normalized MS as a function of temperature in marbled spinefoot and salema.

The curves shown in Fig. 2 describe the relationship between MS and water temperature for both native and invasive species. The values of the empirical parameters obtained by the fitting procedure are shown in Table 1, for each species. Metabolic scope increased as a function of temperature until it reached a maximal value. The optimal temperature for aerobic scope (*T*_opt_) was defined as the temperature at which the MS was maximum. Beyond *T*_opt_, MS decreased at a certain rate until it reached zero. Critical temperature (*T*_crit_) was defined as the temperature at which MS was equal to zero. Metabolic scope curves in the salema and marbled spinefoot were described by different values of *T*_opt_ and *T*_crit_. Metabolic scope increased in the salema up to a maximal value of 709 mg O_2_ kg^−1^ h^−1^ at 21.8°C (i.e. *T*_opt_), whereas at higher temperatures MS decreased to zero at *T* = 28.7°C (i.e. *T*_crit_). In line with their *T*_crit_, none of the salema individuals survived at the acclimation temperatures of 29 and 32°C. In the marbled spinefoot, MS increased up to a maximal value of 850 mgO_2_ kg^−1^ h^−1^ at 29.1°C (i.e. *T*_opt_), whereas at higher temperatures it decreased to zero at *T* = 37.5°C (i.e. *T*_crit_). The normalized MS as a function of water temperature is shown in Fig. 2b for both species.

**Table 1.** List of the parameters and corresponding values used to estimate Eq. (2) and (3).

Parameters	Units	Values for *S. salpa*	Values for *S. rivulatus*
*a*	mg O_2_ kg^−1^ h^−1^	190.2	176.7
*b*	°C^−1^	0.067	0.050
*ω*		1.4	1.3
*δ*	mg O_2_ kg^−1^ h^−1^	6931.1	6762.2
*T*_m_	°C	32.8	42.7
*T*_act_	°C	24.3	32.2

The accuracy of the computed empirical function was verified by comparing the MS obtained from the empirical measurements of MMR and SMR, carried out during the whole set of laboratory experiments in both fish species, and the predicted ones (MS_pre_). A linear regression analysis based on equation (1) was performed (forcing the regression line through the origin), and the following significant relationship was found:

(7) $${\text{MS}}_{\mathrm{obs}}=(1.01\pm 0.026)\times {\text{MS}}_{\mathrm{pre}},$$

with *P* < 0.0001, *N* = 106 and *r*^2^ = 0.80. The linear relationship obtained was characterized by a slope not significantly different from one (*P* = 0.50), indicating an acceptable level of accuracy in the empirical model when predicting MS in relationship to *T*.

### Thermal habitat predictions

The model results were analysed to obtain the variability of the daily average temperature at the basin and regional levels for two climatological windows, representative of the decade 2000–2009 (present-day projection) and the decade 2040–2049 (future scenario), respectively. At the basin scale, a general rise in average temperature up to 1°C with an average increase of around 0.6°C was found between the present-day projection and the future scenario. The highest increase in temperature, corresponding to 1.8°C, was found in the Northern Adriatic Sea during the winter period. Lower increments, between 0.3 and 0.7°C, were computed for the central and deeper areas of the eastern and western sub-basins, throughout the whole year. At the regional scale, in the Sicilian Channel, the surface temperature varied considerably from the southern, warmer part of the channel to the northern, colder one. According to the present-day projection results, the southern area was characterized by high variability in surface temperature [with the lowest values during the winter (between 14.5 and 20.7°C, monthly averaged temperatures) and the highest values in summer (between 21.5 and 30.1°C, monthly averaged temperatures)]. This was especially true in the shallow water banks in front of Tunisia. For this area, the increase in surface temperature between present-day projection and future scenario ranged between 0.3 and 1.3°C, with the maximal increment found during the autumn and the minimal increment during the spring. The northern area was characterized by low variability of the surface temperature. This was due both to the presence of Atlantic Water, which restrains the seasonal changes of temperature along the Sicilian coastal areas, and to the higher latitudes. The lowest temperatures were computed during the winter (ranging between 13.1 and 18.5°C, monthly averaged temperatures) with maximal and minimal values found in December and January, respectively. During the summer, the computed monthly averaged temperature varied between 17.5 and ∼26.5°C, with the maximum found in August and the minimum in June. Along with the lower variability, the results obtained by the future scenario revealed a marked increase in the surface temperature in the southern part of the basin. The increase in temperature varied between 0.2 and 1.5°C, with the maximum value during the autumn and the minimum in the spring.

### Physiological–oceanographic coupling at basin scale

**Figure 3:**
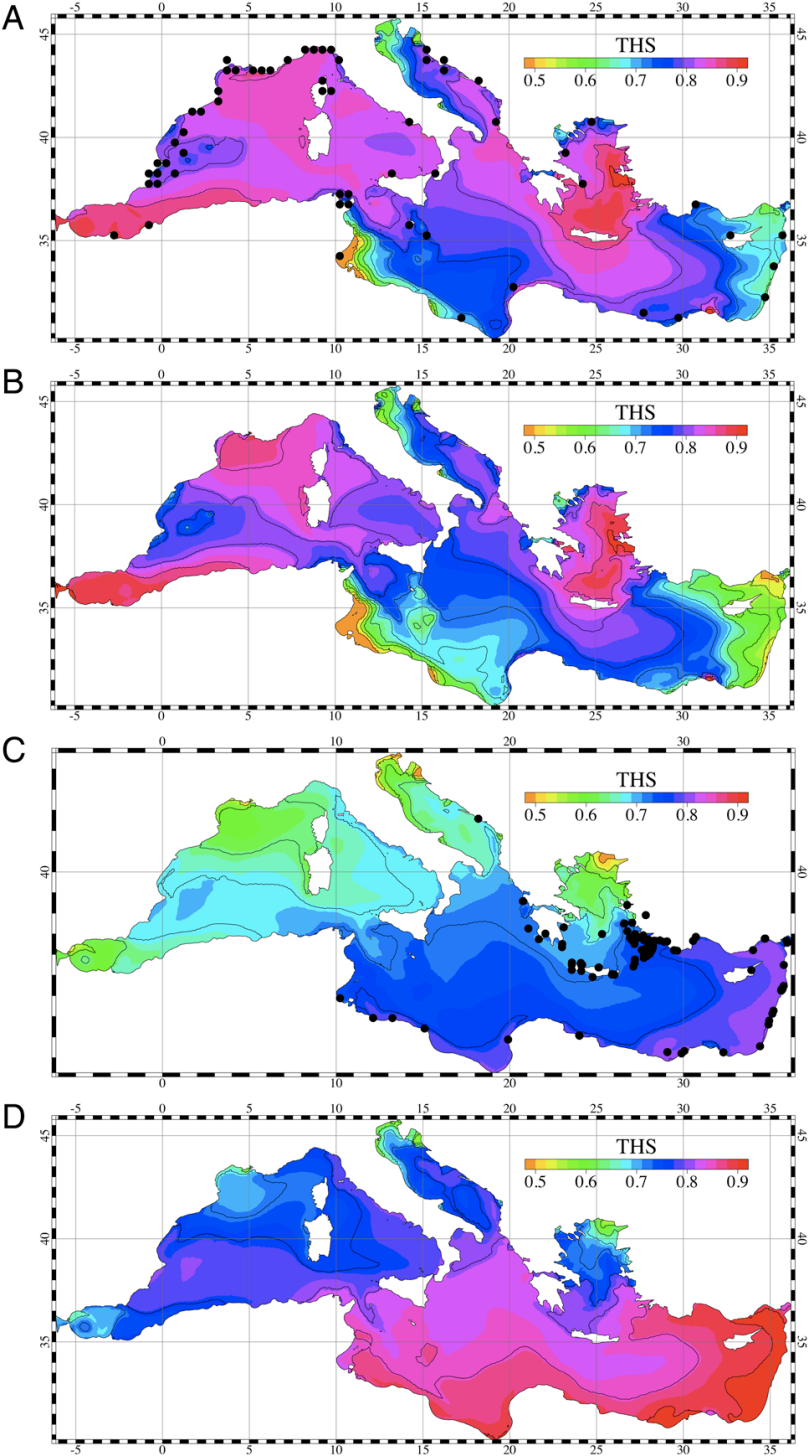
Thermal habitat suitability (THS) computed for the whole Mediterranean Sea from the basin-scale model results. (A) Thermal habitat suitability of the salema computed from the present-day simulation results. (B) Thermal habitat suitability of the salema computed from simulation results of the future scenario. (C) Thermal habitat suitability of the marbled spinefoot computed from present-day projection results. (D) Thermal habitat suitability of the marbled spinefoot computed from the simulation results of the future scenario. Black dots represent the sites where the fish species were observed.

At basin scale, the MS and NMS of the two fish species were computed using surface water temperature data obtained by a simulation run (MOD1) for the present-day projection and the future scenario. The distributions of the THS obtained from the present-day projection and the future simulated scenario are shown in Fig. 3. The present-day projection results show a clear distinction between the values obtained for the eastern and the western parts of the basin, with high THS values found in the former. This is in line with the existing data on distribution, which suggest that the current thermal habitat of the eastern basin is suitable for the marbled spinefoot, while in the western basin the THS values are as low as 50–60% of the maximum. In contrast, most of the Mediterranean Sea shows relatively high THS for the salema*,* in line with the current widespread distribution of this species (data for salema, www.fishbase.org; data for the marbled spinefoot, Azzurro *et al.*, 2013b). Based on the THS isoline including 95% of the current distribution of marbled spinefoot and salema, a threshold below which the thermal habitat conditions become unsuitable in each species (*T*_MS_) was identified as 0.62 of the maximal THS. The future scenario results show an increase in the extension of areas thermally suitable for the marbled spinefoot, such that virtually all of the Mediterranean Sea shows THS values >62% of the maximum. Conversely, for the salema, the thermally suitable area decreases in size, with large areas showing values around 50–60% of the maximal THS along the Lebanese and Tunisian–Libyan coasts.

### Physiological–oceanographic coupling at regional scale

**Figure 4:**
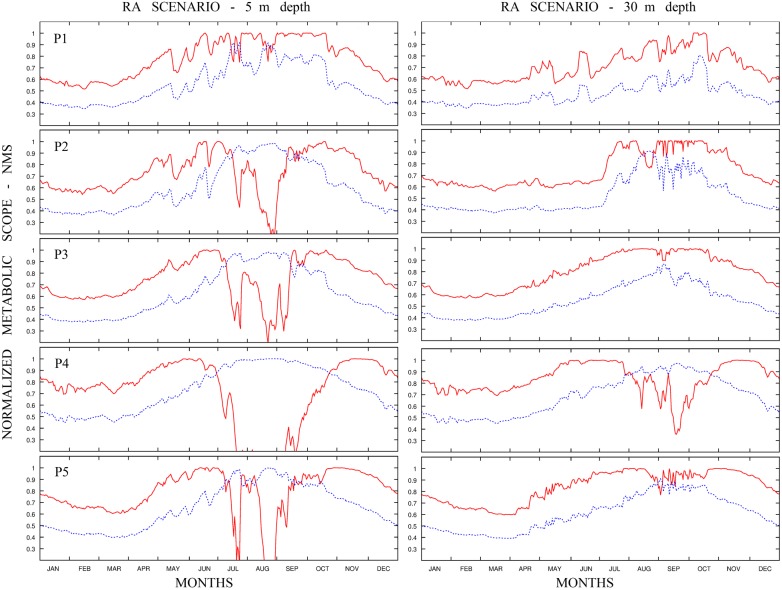
Present-day projection. Temporal variability of the daily normalized MS computed for salema (red line) and for marbled spinefoot (blue line) at different depths for the five selected sites in the Sicilian Channel, throughout the year (averaged over 10 years). Left panel refers to results at 5 m depth and right panel at 30 m depth.

**Figure 5:**
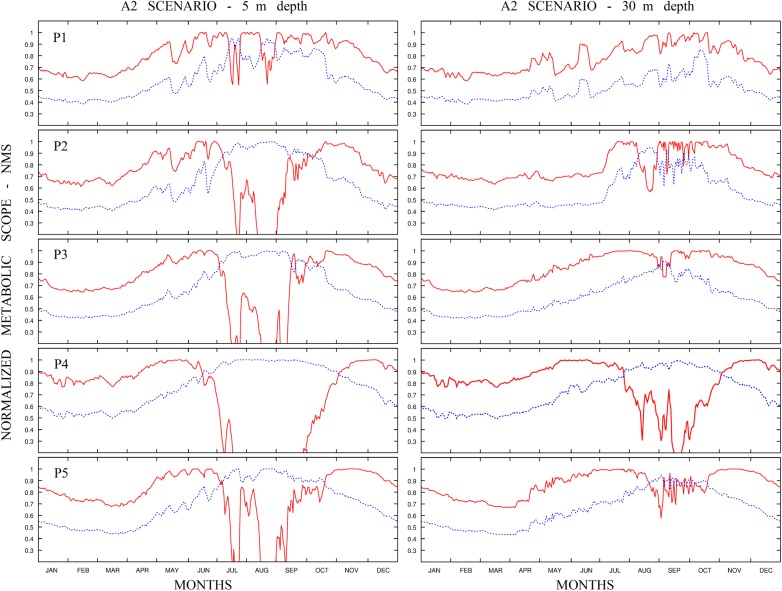
Future scenario. Temporal variability of the daily normalized MS computed for the salema (red line) and for the marbled spinefoot (blue line) at different depths for the five selected sites in the Sicilian Channel, throughout the year (averaged over 10 years). Left panel refers to the results at 5 m depth and right panel at 30 m depth.

**Figure 6:**
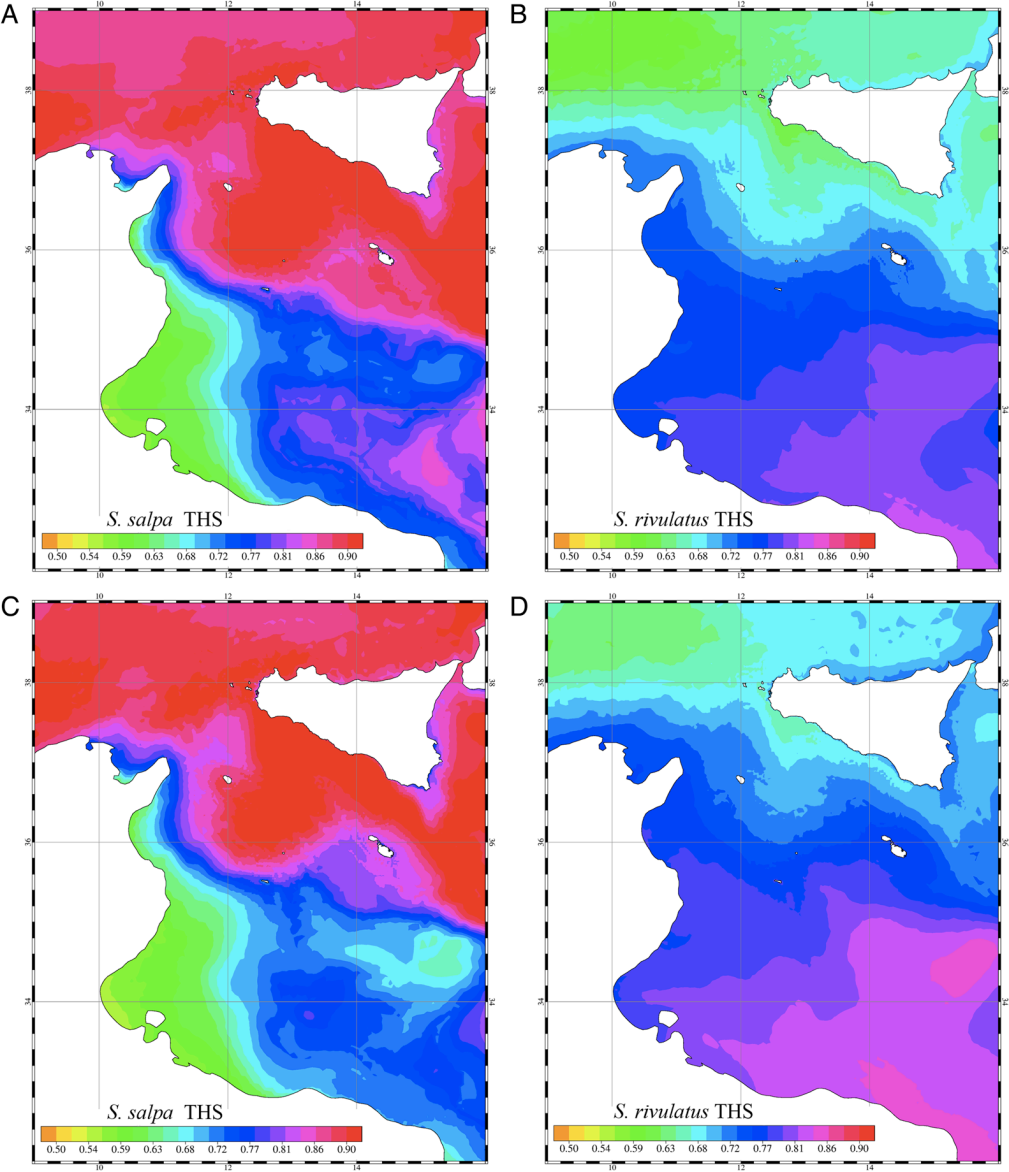
Thermal habitat suitability (THS) of salema and marbled spinefoot computed for the present-day projection and the future scenarios. (A) and (B) Thermal habitat suitability of salema and marbled spinefoot, respectively, computed from present-day projection simulation results. (C) and (D) Thermal habitat suitability of salema and marbled spinefoot, respectively, computed from future scenario simulation results.

**Figure 7:**
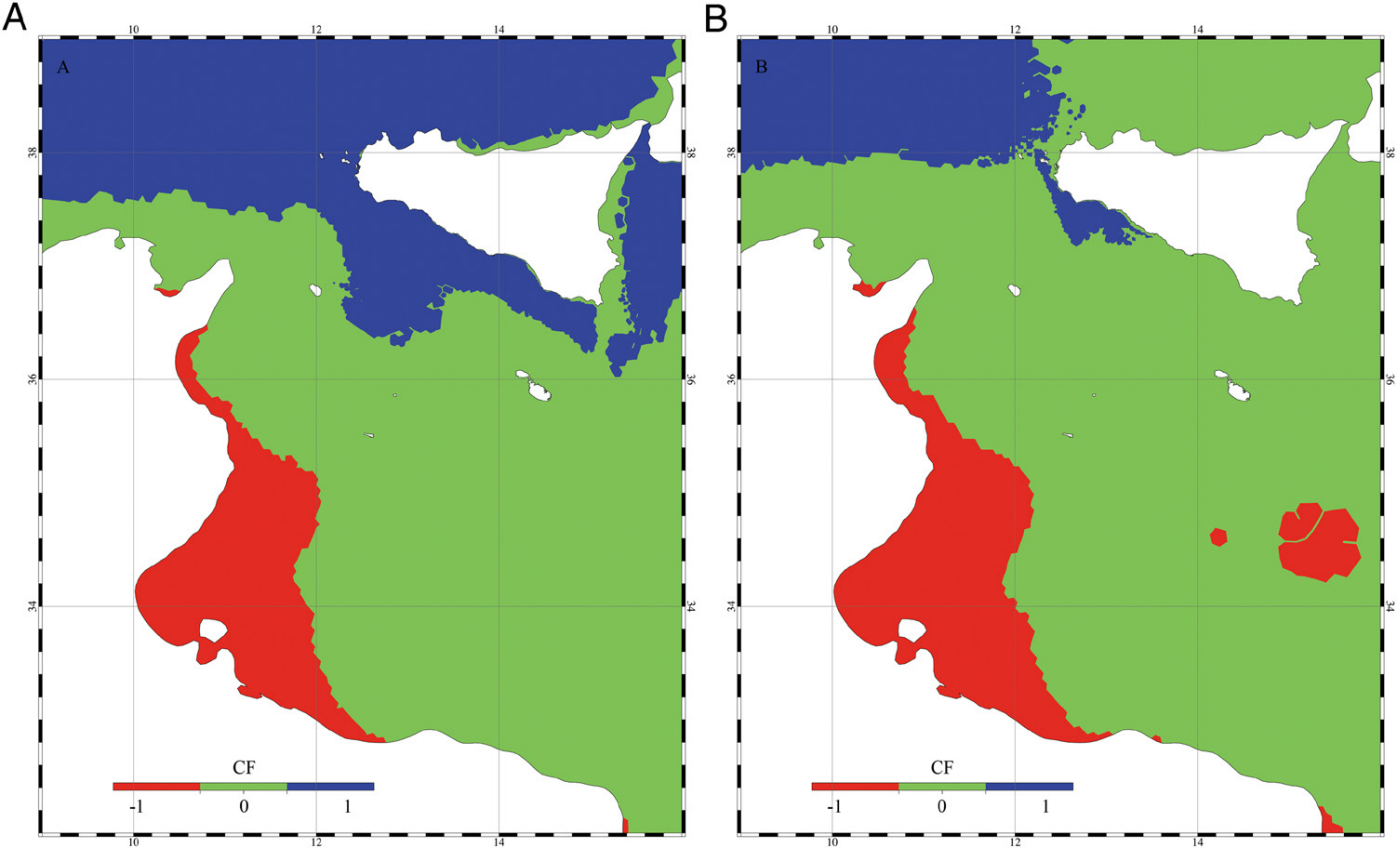
(A) Coexistence factor (CF) computed for the present-day projection. (B) Coexistence factor computed for the future scenario.

The numerical approach used at the regional scale allowed the reproduction of the hydrodynamic conditions in more detail. The computation of the NMS for the two fish species was carried out at different depths (i.e. between 5 and 30 m), within the depth range of distribution of the two species [i.e. salema 0–70 m; marbled spinefoot 0–60 m (Louisy, 2005)]. The time series of NMS of the two species computed at five locations selected in the Sicilian Channel are shown in Fig. 4 for the present-day projection and Fig. 5 for the future scenario. For the present-day projection, the NMS of the salema showed a strong decrease in the summer. Conversely, the pattern of NMS for the marbled spinefoot showed an increase in NMS during the summer months, together with a higher overall NMS in southern than in northern sites. The future scenario showed a general increase of NMS in the marbled spinefoot in all stations and at all depths. In contrast, the NMS of the salema showed a general reduction, especially during the summer. The spatial distributions of THS for the present-day projection and the future scenario are shown in Fig. 6. For the salema, the areas of high THS value tended to decrease in size in the future scenario compared with the present-day projection, especially in the southern sites, while the opposite is true for the marbled spinefoot. This trend is confirmed by comparing the coexistence factor between the present-day projection and the future scenario (Fig. 7). The future scenario showed an increase of the areas where the CF < 0 (i.e. areas where THS of the marbled spinefoot is higher than that of the salema) and where CF = 0 (i.e. areas where THS is favourable for both fish) and a decrease of areas with CF > 0 (i.e. areas where THS of the salema is higher than that of the marbled spinefoot). Note that where CF = 0, the QMS of both fish species were always equal to one so that no cases of THS unfavourable for both fish species were found. Specifically, when comparing the present-day projection with the future scenario, the area of suitability increased from 4 to 7% for CF < 0 and from 66 to 82% for CF = 0, while it decreased from 30 to 11% for CF > 0.

## Discussion

Our work represents the first attempt at comparing the physiological performance of a native fish species with that of its invasive counterpart integrated with an oceanographic modelling approach. For the present-day scenario, our basin-scale model showed higher THS for the invasive marbled spinefoot in the Eastern compared with the Western Mediterranean Sea. According to our basin-scale model, over the next 40 years the THS of the marbled spinefoot is expected to increase in the Western Mediterranean Sea, reaching values currently found in the Eastern Mediterranean, where the species is already well established (Golani, 1993).

The relationship between aerobic scope and temperature is in agreement with previous work that explored the effects of temperature on the physiological performance of siganids (Saoud *et al.*, 2008). Saoud and colleagues found that growth rate [a variable known to be affected by aerobic scope (Pörtner and Knust, 2007; Khan *et al.*, 2014)] was maximized in marbled spinefoot kept at 27°C (Saoud*, et al.*, 2008). However, no data are available on the effect of temperature on the physiological performance of the salema*.* Interestingly, the metabolic scope of *Mugil cephalus* (a coastal fish species commonly found near the surface within a shallow depth range; Louisy, 2005) show similar, though slightly higher, values *T*_opt_ to those found here for the salema, i.e. ∼25°C (Cucco *et al.*, 2012a).

The thermal habitat suitability of both marbled spinefoot and salema largely matched the current distribution patterns of the two species. Currently, the marbled spinefoot occupies most of the Eastern Mediterranean but has not yet entered the western basin. Interestingly, the only part of the eastern basin where the marbled spinefoot has not been reported is the northern Aegean Sea (Zenetos *et al.*, 2010; Golani *et al.*, 2002; Vergés *et al.*, 2014b). Our model showed relatively low THS values for this area, similar to those found in the Western Mediterranean Sea. The competitive superiority of the marbled spinefoot in the Eastern Mediterranean Sea when compared with salema may also be due to its greater adaptability to fluctuating environmental conditions (Bariche *et al.*, 2004). Juvenile marbled spinefoot were found to be highly tolerant to variations in temperature and salinity, oxygen deficiency and overcrowding (Ben Tuvia *et al.*, 1973; Popper and Gundermann, 1975).

According with the predictions based on the IPCC scenarios (IPCC, 2013), over the next 40 years the thermal properties of the Western Mediterranean could become suitable for the marbled spinefoot, if it reaches similar temperature values currently occurring in the Eastern Mediterranean. At the same time, large sectors of the eastern basin (e.g. Lebanese and Libyan coast) could become thermally unsuitable for the salema, which could progressively restrict its distribution to the western basin. Hence, the marbled spinefoot is expected to move into the western basin and compete with the salema. In the eastern basin, we predict that it will slowly replace the salema in several areas, as has already happened in the coastal waters of Lebanon (Bariche *et al*., 2004). The salema was still an abundant species in the 1930s, when a modest population of marbled spinefoot occurred along the coast of Lebanon and Syria. The marbled spinefoot population has increased significantly since then, and competition between the two species was noted in the 1960s in the same area (reviewed by Kalogirou *et al.*, 2012). Considering the absence of salema along the coast of Lebanon in recent observations (Bariche *et al.*, 2004), the displacement process of the native fish can be estimated to have occurred within the past five to seven decades.

The documented spread of the marbled spinefoot in the Eastern Mediterranean has led to important consequences for the composition and structure of subtidal benthic communities. This has caused algal forests to turn into ‘barrens’, where canopy algae abundance, benthic biomass and species richness are dramatically reduced. Recent work has found functional differences in the feeding behaviour of the salema and the marbled spinefoot (Vergés *et al.*, 2014b). Although the salema showed a higher overall feeding rate in terms of biomass consumption, this temperate fish species feeds exclusively on adult microalgae, while the marbled spinefoot feeds on both adult microalgae and epilithic algal matrix, which, in most cases, contains microalgal recruits. Therefore, the reduction in the epilithic algal matrix biomass, caused by the feeding habits of the tropical marbled spinefoot, can severely affect the biomass and biodiversity of the temperate reefs (Vergés *et al.*, 2014b). This does not only cause a direct reduction in the algal abundance and diversity, but also implies a loss of critical habitat for understory algae and many invertebrates that live in the algal canopies (Vergés *et al.*, 2014b), which are the prey for the majority of carnivorous fish species.

The Sicilian Channel area may be considered the ‘door’ to the western basin; therefore, we have focused our attention on this area, for which a high-resolution model was run. Our results suggest that the thermal habitat of the southern part of the Sicilian Channel will be increasingly more suitable for the marbled spinefoot than for the salema (Figs 6 and 7), and a population increase of the invasive species in these sectors (which include a large part of the Tunisian coasts) could represent a significant step for the westward progress of the invasion. In areas such as Malta and south-eastern Sicily, the thermal suitability for marbled spinefoot and salema is predicted to reach similar values by 2050 (Fig. 7). Moreover, the northern part of the Sicilian Channel was predicted to become thermally suitable for both species (Fig. 6). Therefore, the invasion of the marbled spinefoot could potentially expand northward as a result of warming seawater conditions. Conversely, in south-western Sicily, THS could remain relatively unsuitable for the marbled spinefoot.

Our findings could have implications for a variety of questions in ecology, invasive biology and climate change (Pörtner and Peck, 2010; Jørgensen *et al.*, 2012). To predict the invasive potential of Lessepsian fish species, biotic processes such as interspecific competition (Azzurro *et al.*, 2014) need to be taken into account, in addition to the direct response to abiotic variables (i.e. temperature increase, induced by global warming; Killen *et al.*, 2013). The present model is, admittedly, simplistic. In a warming scenario, species distribution shifts are likely to depend on a number of factors in addition to metabolic scope; these include food availability, predation and competition, as well as other abiotic factors, such as oxygen levels and salinity. However, temperature changes in the Mediterranean Sea could act as a critical factor influencing invasion success by affecting the physiological mechanisms responsible for invader superiority in a global warming scenario (Ben Rais Lasram *et al.*, 2010; Albouy *et al.*, 2012, 2013). Further work on our model could be directed at including additional variables, such as food availability, thereby increasing the predictive power of the model. Furthermore, a useful addition to the present study would be the individual-based analyses of species' realized functional niches, aimed at quantifying the possible functional overlap between the marbled spinefoot and the salema in various thermal conditions. An example is found in species-specific microhabitat utilization patterns of herbivores (Brandl and Bellwood, 2014). In addition, behavioural work could be performed to test the possibility that competitive displacement may exacerbate the effect of temperature on fish performance, leading to habitat relocation of the ‘loser’ species, as found in other competing fish pairs (Milazzo *et al.*, 2013).

At present, different models have been used to assess fish distribution and abundance. One of the most common is the bio-envelope model, which examines the relationship between key climatic variables and species distributions, mostly based on historical distributional data (Ben Rais Lasram *et al.*, 2010; Albouy *et al.*, 2012, 2013). Here, we propose the use of a physiology-based model to predict fish distribution. This type of model relies on solid, reproducible laboratory experiments, and uses metabolic scope as a proxy for the ecosystem thermal suitability in different climate scenarios. In this respect, it provides an advance in the use of mechanistic, cause–effect approaches for understanding the effect of environmental changes on habitat suitability. This approach might provide a physiologically driven tool for understanding and predicting the distribution and relative abundance of marine fish.

## Funding

This work was supported by the European Community's Seventh Framework Programme (FP7/2007–2013) under Grant Agreement No. 266445 for the project Vectors of Change in Oceans and Seas Marine Life, Impact on Economic Sectors (VECTORS) and by the RITMARE Project, funded by the Italian Ministry of Education, Universities and Research.

## References

[COU059C1] Albouy C Guilhaumon F Araújo MB Mouillot D Leprieur F (2012) Combining projected changes in species richness and composition reveals climate change impacts on coastal Mediterranean fish assemblages. Glob Change Biol 18: 2995–3003. 10.1111/j.1365-2486.2012.02772.x 28741816

[COU059C2] Albouy C Guilhaumon F Leprieur F Ben Rais Lasram F Somot S Aznar R Velez L Le Loc'h F Mouillot D (2013) Projected climate change and the changing biogeography of coastal Mediterranean fishes. J Biogeogr 40: 534–547.

[COU059C3] Astraldi M Balopoulos S Candela J Font J Gacic M Gasparini GP Manca B Theocharis A Tintoré J (1999) The role of straits and channels in understanding the characteristics of Mediterranean circulation. Progr Oceanogr 44: 65–108.

[COU059C4] Azzurro E Andaloro F (2014) A new settled population of the lessepsian migrant *Siganus luridus* (Pisces: Siganidae) in Linosa Island—Sicily Strait. J Mar Biol Assoc UK 84: 819–821.

[COU059C5] Azzurro E Fanelli E Mostarda E Catra M Andaloro F (2007) Resource partitioning among early colonizing *Siganus luridus* and native herbivorous fish in the Mediterranean: an integrated study based on gut-content analysis and stable isotope signatures. J Mar Biol Assoc UK 87: 991–998.

[COU059C6] Azzurro E Soto S Garofalo G Maynou F (2012) *Fistularia commersonii* in the Mediterranean sea: invasion history and distribution modeling based on presence-only records. Biol Invas 5: 977–990.

[COU059C7] Azzurro E La Mesa G Fanelli E (2013a) The rocky-reef fish assemblages of Malta and Lampedusa islands (Strait of Sicily, Mediterranean Sea): a visual census study in a changing biogeographical sector. J Mar Biol Assoc UK 93: 2015–2026.

[COU059C8] Azzurro E Soto S Bariche M Fanelli W Maynou F (2013b) Exotic fish species in the Mediterranean Sea: analysis of occurrence records. Rapp Comm Int Mer Médit 40: 508.

[COU059C9] Azzurro E Tuset V Lombarte A Maynou F Simberloff D Pérez A Sole R (2014) External morphology explains the success of biological invasions. Ecol Lett 17: 1455–1463. 25227153 10.1111/ele.12351

[COU059C10] Bariche M Letourneur Y Harmelin-Vivien M (2004) Temporal fluctuations and settlement patterns of native and Lessepsian herbivorous fishes on the Lebanese coast (eastern Mediterranean). Environ Biol Fishes 70: 81–90.

[COU059C11] Belmaker J Parravicini V Kulbicki M (2013) Ecological traits and environmental affinity explain Red Sea fish introduction into the Mediterranean. Glob Change Biol 19: 1373–1382. 10.1111/gcb.12132 23505033

[COU059C12] Ben Rais Lasram F Guilhaumon F Albouy C Somot S Thuiller W Mouillot D (2010) The Mediterranean Sea as a ‘cul-de-sac’ for endemic fishes facing climate change. Glob Change Biol 16: 3233–3245.

[COU059C13] Ben Tuvia A Kissil GW Popper D (1973) Experiments in rearing rabbitfish *Siganus rivulatus* in sea water. Aquaculture 1: 359–364.

[COU059C14] Blois JL Zarnetske PL Fitzpatrick MC Finnegan S (2013) Climate change and the past, present, and future of biotic interactions. Science 341: 499–504. 23908227 10.1126/science.1237184

[COU059C15] Brandl SJ Bellwood DR (2014) Individual-based analyses reveal limited functional overlap in a coral reef fish community. J Anim Ecol 33: 421–430. 10.1111/1365-2656.12171 24164060

[COU059C16] Burrows MT Schoeman DS Buckley LB Moore P Poloczanska ES Brander KM Brown C Bruno JF Duarte CM Halpern BS (2011) The pace of shifting climate in marine and terrestrial ecosystems. Science 334: 652–655. 22053045 10.1126/science.1210288

[COU059C17] Chabot D Claireaux G (2008) Environmental hypoxia as a metabolic constraint on fish: the case of Atlantic cod, *Gadus morhua*. Mar Pollut Bull 57: 287–294. 18508091 10.1016/j.marpolbul.2008.04.001

[COU059C18] Cucco A Umgiesser G Ferrarin C Perilli A Canu DM Solidoro C (2009) Eulerian and lagrangian transport time scales of a tidal active coastal basin. Ecol Model 220: 913–922.

[COU059C19] Cucco A Sinerchia M Lefrancois C Magni P Ghezzo M Umgiesser G Perilli A Domenici P (2012a) A metabolic scope based model of fish response to environmental changes. Ecol Model 237–238: 132–141.

[COU059C20] Cucco A Sinerchia M Ribotti A Olita A Fazioli L Sorgente B Perilli A Borghini M Schroeder K Sorgente R (2012b) A high-resolution real-time forecasting system for predicting the fate of oil spills in the Strait of Bonifacio (western Mediterranean Sea). Mar Pollut Bull 64: 1186–1200. 22498317 10.1016/j.marpolbul.2012.03.019

[COU059C21] Fry FE (1971) The effect of environmental factors on the physiology of fish. In Hoar WS Randall DJ, eds, Fish Physiology, Vol. VI Academic Press, New York, NY, USA, pp 1–98.

[COU059C22] Galil BS (2009) Taking stock: inventory of alien species in the Mediterranean Sea. Biol Invas 11: 359–372.

[COU059C23] Golani D (1993) Trophic adaptations of Red Sea fishes to the eastern Mediterranean environment – review and new data. Israel J Zool 39: 391–402.

[COU059C24] Golani D Orsi-Relini L Massuti E Quignard JP (2002) CIESM Atlas of Exotic Species in the Mediterranean. Vol 1 Fishes, CIESM, Monaco, 256 p.

[COU059C25] Harley CDG Hughes AR Hultgren KM Miner BG Sorte CJB Thornber CS Rodriguez LF Tomanek L Williams SL (2006) The impacts of climate change in coastal marine systems. Ecol Lett 9: 228–241. 16958887 10.1111/j.1461-0248.2005.00871.x

[COU059C26] Hiddink JG Ben Rais Lasram F Cantrill J Davies AJ (2012) Keeping pace with climate change: what can we learn from the spread of Lessepsian migrants? Glob Change Biol 18: 2161–2171.

[COU059C27] Holt JT James ID (2001) An *s* coordinate density evolving model of the northwest European continental shelf: 1. Model description and density structure. J Geophys Res 106: 14015–14034.

[COU059C28] IPCC (2013) Climate Change 2013: the Physical Science Basis. Working group 1 contribution to the fifth assessment report of the International Panel on Climate Change, International Panel on Climate Change, Cambridge, UK and New York, NY, USA.

[COU059C29] Jørgensen C Peck MA Antognarelli F Azzurro E Burrows T Cheung WWL Cucco A Holt RE Huebert KB Marras S (2012) Conservation physiology of marine fishes: advancing the predictive capacity of models. Biol Lett 8: 900–903. 22859560 10.1098/rsbl.2012.0609 PMC3497128

[COU059C30] Jungclaus JH Keenlyside N Botzet M Haak H Luo J-J Latif M Marotzke J Mikolajewicz U Roeckner E (2006) Ocean circulation and tropical variability in the coupled model echam5/mpi-om. J Climate 19: 3952–3972.

[COU059C31] Kalogirou S Azzurro E Bariche M (2012) The ongoing shift of Mediterranean coastal fish assemblages and the spread of non-indigenous species. In Lameed AG ed, Biodiversity Enrichment in a Diverse World. InTech, Rijeka, Croatia, pp 263–280.

[COU059C32] Khan JR Pether S Bruce M Walker SP Herbert NA (2014) Optimum temperatures for growth and feed conversion in cultured hapuku (*Polyprion oxygeneios*)—is there a link to aerobic metabolic scope and final temperature preference? Aquaculture 430: 107–113.

[COU059C33] Killen SS Marras S McKenzie DJ (2011) Fuel, fasting, fear: routine metabolic rate and food deprivation exert synergistic effects on risk taking in individual juvenile European seabass. J Anim Ecol 80: 1024–1033. 21790592 10.1111/j.1365-2656.2011.01844.x

[COU059C34] Killen SS Marras S Ryan MR Domenici P McKenzie DJ (2012) A relationship between metabolic rate and risk-taking behaviour is revealed during hypoxia in juvenile European sea bass. Funct Ecol 26: 134–143.

[COU059C35] Killen SS Marras S Metcalfe NB McKenzie DJ Domenici P (2013) Environmental stressors alter relationships between physiology and behaviour. Trends Ecol Evol 28: 651–658. 23756106 10.1016/j.tree.2013.05.005

[COU059C36] Langmead O McQuatters-Gollop A Mee LD (Eds) (2007) European Lifestyles and Marine Ecosystems: Exploring Challenges for Managing Europe's Seas. University of Plymouth Marine Institute, Plymouth, UK.

[COU059C37] Lefrançois C Claireaux G (2003) Influence of ambient oxygenation and temperature on metabolic scope and scope for heart rate of the sole (*solea solea*). Mar Ecol Prog Ser 259: 273–284.

[COU059C38] Louisy P (2005) Guide d'Identification des Poisson Marins Europe et Mediterranee. Les Editions Eugen Ulmer, Paris, France.

[COU059C39] Malanotte-Rizzoli P (2001) Currents systems in the Mediterranean Sea. Steele J Thorpe S Turekian K, Encyclopedia of Ocean Sciences. Elsevier, Oxford, UK, pp 605–612.

[COU059C40] Manzella GMR Gasparini GP Astraldi M (1988) Water exchange between the eastern and western Mediterranean through the Strait of Sicily. Deep-Sea Res Part A Oceanogr Res Papers 35: 1021–1035.

[COU059C41] Marras S Claireaux G McKenzie DJ Nelson JA (2010) Individual variation and repeatability in aerobic and anaerobic swimming performance of European sea bass, *Dicentrarchus labrax*. J Exp Biol 213: 26–32. 20008358 10.1242/jeb.032136

[COU059C42] Marras S Killen SS Domenici P Claireaux G McKenzie DJ (2013) Relationships among traits of aerobic and anaerobic swimming performance in individual European sea bass, *Dicentrarchus labrax*. PLoS ONE 8: e72815. 24019879 10.1371/journal.pone.0072815 PMC3760853

[COU059C43] Marullo S Buongiorno Nardelli B Guarracino M Santoleri R (2007) Observing the Mediterranean Sea from space: 21 years of Pathfinder-AVHRR sea surface temperatures (1985 to 2005). Re-analysis and validation. Ocean Sci 3: 299–310.

[COU059C44] Milazzo M Mirto S Domenici P Gristina M (2013) Climate change exacerbates interspecific interactions in sympatric coastal fishes. J Anim Ecol 82: 468–477. 23039273 10.1111/j.1365-2656.2012.02034.x

[COU059C45] Perry AL Low PJ Ellis JR Reynolds JD (2005) Climate change and distribution shifts in marine fishes. Science 308: 1912–1915. 15890845 10.1126/science.1111322

[COU059C46] Popper D Gundermann N (1975) Some ecological and behavioral aspects of siganids population in the Red Sea and the Mediterranean coast of Israel in relation to their suitability for aquaculture. Aquaculture 6: 127–141.

[COU059C47] Por FD (1990) Lessepsian migration. An appraisal and new data. Bulletin de l'Institut Oceanographique (Monaco) 7: 1–10.

[COU059C48] Pörtner HO Farrell AP (2008) Physiology and climate change. Science 322: 690–692. 18974339 10.1126/science.1163156

[COU059C49] Pörtner HO Knust R (2007) Climate change affects marine fishes through the oxygen limitation of thermal tolerance. Science 315: 95–97. 17204649 10.1126/science.1135471

[COU059C50] Pörtner HO Peck MA (2010) Climate change effects on fishes and fisheries: towards a cause-and-effect understanding. J Fish Biol 77: 1745–1779. 21078088 10.1111/j.1095-8649.2010.02783.x

[COU059C51] Quignard JP Tomasini JA (2000) Mediterranean fish biodiversity. Biologia Marina Mediterranea 7: 1–66.

[COU059C52] Roche DG Binning SA Bosiger Y Johansen JL Rummer JL (2013) Finding the best estimates of metabolic rates in a coral reef fish. J Exp Biol 216: 2103–2110. 23470659 10.1242/jeb.082925

[COU059C53] Roeckner E Brokopf R Esch M Giorgetta M Hagemann S Kornblueh L Manzini E Schlese U Schulzweida U (2006) Sensitivity of simulated climate to horizontal and vertical resolution in the ECHAM5 atmosphere model. J Climate 19: 3771–3791.

[COU059C54] Sala E Kizilkaya Z Yildirim D Ballesteros E (2011) Alien marine fishes deplete algal biomass in the eastern Mediterranean. PLoS ONE 6: e17356. 21364943 10.1371/journal.pone.0017356 PMC3043076

[COU059C55] Saoud IM Mohanna C Ghanawi J (2008) Effects of temperature on survival and growth of juvenile spinefoot rabbitfish (*Siganus rivulatus*). Aquac Res 39: 491–497.

[COU059C56] Schembri PJ Deidun A Falzon MA (2012) One *Siganus* or two? On the occurrence of *Siganus luridus* and *Siganus rivulatus* in the Maltese Islands. Mar Biodivers Rec 5: e71.

[COU059C57] Seitzinger SP Harrison JA Dumont E Beusen AHW Bouwman AF (2005) Sources and delivery of carbon, nitrogen, and phosphorus to the coastal zone: an overview of global Nutrient Export from Watersheds (NEWS) models and their application. Glob Biochem Cycles 19: GB4S01.

[COU059C58] Somot S Sevault F Déqué M Crépon M (2008) 21st century climate change scenario for the Mediterranean using a coupled atmosphere–ocean regional climate model. Glob Planet Change 63: 112–126.

[COU059C59] Steffensen JF (1989) Some errors in respirometry of aquatic breathers: how to avoid and correct for them. Fish Physiol Biochem 6: 49–59. 24226899 10.1007/BF02995809

[COU059C60] Umgiesser G Bergamasco A (1995). Outline of a primitive equation finite element model. In Rapporti e Studi, Vol. XII Istituto Veneto di Scienze, Lettere ed Arti, Venice, Italy, pp 291–320.

[COU059C61] Umgiesser G Canu D Cucco A Solidoro C (2004) A finite element model for the Venice lagoon. Development, set up, calibration and validation. J Marine Syst 51: 123–145.

[COU059C62] Vergés A Steinberg PD Hay ME Poore AG Campbell AH Ballesteros E Heck KLJ Booth DJ Coleman MA Feary DA (2014a) The tropicalization of temperate marine ecosystems: climate-mediated changes in herbivory and community phase shifts. Proc Biol Sci 281: 20140846. 25009065 10.1098/rspb.2014.0846 PMC4100510

[COU059C63] Vergés A Tomas F Cebrian E Ballesteros E Kizilkaya Z Dendrinos P Karamanlidis AA Spiegel D Sala E (2014b) Tropical rabbitfish and the deforestation of a warming temperate sea. J Ecol 102: 1518–1527.

[COU059C64] Verlaque M (1990) Relations entre *Sarpa salpa* (Linnaeus, 1758) (Teleostean, Sparidae) et les autres poissons brouteurs et le phytobentos algal Mediterraneen. Oceanologica Acta 13: 373–388.

[COU059C65] Zarnetske PL Skelly DK Urban MC (2012) Biotic multipliers of climate change. Science 336: 1516–1518. 22723403 10.1126/science.1222732

[COU059C66] Zenetos A Gofas S Verlaque M Çinar ME García Raso E Bianchi CN Morri C Azzurro E Bilecenoğlu M Froglia C (2010) Alien species in the Mediterranean Sea by 2010. A contribution to the application of European Union's Marine Strategy Framework Directive (MSFD). Part I. Spatial distribution. Mediterranean Mar Sci 11: 381–493.

[COU059C67] Zenetos A Gofas S Morri C Rosso A Violanti D Garcia JE Cinar ME Almogi-Labin A Ates AS Azzurro E (2012) Alien species in the Mediterranean Sea by 2012. A contribution to the application of European Union's Marine Strategy Framework Directive (MSFD). Part 2. Patterns in introduction trends and pathways. Mediterranean Mar Sci 13: 312–336.

